# Mixture of Sodium Hypochlorite and Hydrogen Peroxide on Adhered *Aeromonas hydrophila* to Solid Substrate in Water: Impact of Concentration and Assessment of the Synergistic Effect

**DOI:** 10.1155/2014/121367

**Published:** 2014-03-03

**Authors:** Chrétien Lontsi Djimeli, Antoine Tamsa Arfao, Olive V. Noah Ewoti, Mireille Ebiane Nougang, Marlyse L. Moungang, Geneviève Bricheux, Moïse Nola, Télesphore Sime-Ngando

**Affiliations:** ^1^University of Yaoundé I, Laboratory of General Biology, Hydrobiology and Environment Research Unit, P.O. Box 812, Yaoundé, Cameroon; ^2^Laboratoire “Microorganismes: Génome & Environnement”, UMR CNRS 6023, Université Blaise Pascal, Complexe Scientifique des Cézeaux, 24 avenue des Landais, BP 80026, 63171 Aubière Cedex, France

## Abstract

The synergistic effects of the combined treatments of NaOCl and H_2_O_2_ on the elimination of *A. hydrophila* adhered to polythene under static and dynamic conditions were evaluated. The concentrations 0.1, 0.2, and 0.3‰ NaOCl and 0.5, 1, and 1.5‰ H_2_O_2_ were used. The contact periods were 180, 360, 540, and 720 minutes. The abundance of cells adhered reached 2.47 and 2.27 units (log (CFU/cm²)), respectively, under static and dynamic conditions after action of the mixture of disinfectants, whereas it reached 2.41 and 3.39 units (log (CFU/cm²)) after action of NaOCl and H_2_O_2_ alone, respectively. Increase in the incubation period resulted in a significant decrease in the abundance of cells adhered when the mixture of 0.3‰ NaOCl and 1.5‰ H_2_O_2_ was used (*P* < 0.01). For each cell growth phase, there was a significant difference amongst the mean densities of cells adhered after action of the mixture of disinfectants (*P* < 0.05). Although the Freundlich isotherm parameters relatively varied from one experimental condition to another, the *K*_*f*_ value registered in the exponential growth phase was relatively higher in static state than in dynamic regime; cells adhered under dynamic condition seem more sensitive to the synergistic action than those adhered under static condition.

## 1. Introduction

The drinking water distribution network is a source of disquiet regarding the contamination of water during delivery and regrowth of microorganisms that survive after treatment [1]. It is often the scene of many physicochemical and biological reactions resulting from interactions between disinfectants, pipe walls, and the free and fixed biomass [2]. The presence of natural organic matter provides a food source for bacteria that can colonize the inner walls of distribution pipes, forming biofilms that protect and support the growth of microorganisms, some of which are associated to hostile effect on human health [1] and others through their interactions with disinfectants and pipe walls are sometimes the cause of the deterioration of the organoleptic properties of the water supply [2, 3].

In recent years, World Health Organization recognizes *A. hydrophila* as an opportunistic pathogen, implicated as a pathogenic agent in gastroenteritis, septicemia, cellulitis, colitis, meningitis, and respiratory infections [4–6]. To prevent bacterial regrowth, a residual of a disinfectant is maintained in the water distribution network. Previous work has shown that the bacterium *A. hydrophila* is widespread in the environment, especially in water intended for human consumption [7, 8]. Its concentration can sometimes reach 10^2^ CFU/mL at the outlet of treatment plants for drinking water. This concentration may be higher in networks of drinking water distribution due to the growth of *A. hydrophila* on biofilms [7, 9].

The ingestion of water or contaminated food is the common way of progress in the case of *Aeromonas* infection [10]. Numerous studies have been conducted in view of highlighting the inactivation of various waterborne pathogens by various disinfectants, including sodium hypochlorite, hydrogen peroxide, ozone, and chlorine dioxide [11].

The mixture of NaOCl and H_2_O_2_ in water resulted in a redox reaction which gave the following equations [12].

H_2_O_2_/H_2_O: 1,77 v and ClO_2_^−^/ClO^−^: 0,66 v

(1) $$\begin{array}{c} {\text{ClO}}^{-}+2{\text{HO}}^{-}⟶{{\text{ClO}}_{2}}^{-}+{\text{H}}_{2}\text{O}+2{\text{e}}^{-} \end{array}$$

(2) H2O2+2H++2e−⟶2H2O

(3) (1)  and  (2):ClO−+H2O2+2HO−+2H+⟶ClO2−+3H2O

(4) ClO−+H2O2+2H2O⟶ClO2−+3H2O

(5) ClO−+H2O2⟶ClO2−+H2O

(6) Na++ClO−+H2O2⟶Na++ClO2−+H2O

(7) NaClO+H2O2⟶NaClO2+H2O

NaClO_2_ is a very unstable compound that gives NaCl + ^1^O_2_ (singlet oxygen). It resulted in

(8) NaClO+H2O2⟶NaCl+O21+H2O

The reaction between these disinfectants produces singlet oxygen (^1^O_2_), which is a powerful oxidant that rapidly kills bacterial cells. Singlet oxygen short lifespan (100 nanoseconds in lipid media and 50 nanoseconds in the cytoplasm) can diffuse a short distance and react with certain amino acids leading to structural and functional alteration of the membrane causing lipoperoxidation [13]. Less data are available on the bacterial behavior or bacterial metabolism when both disinfectants are dissolved in water at the same time. Less information are also available on the cell survival with respect to the both disinfectants concentrations.

Most studies carried out so far provided some information on the doses of disinfectants and adequate contact duration period to effectively control pathogens of public health importance that are commonly used to develop regulations and strategies treatment. Chemical disinfectants cause lethal or nonlethal changes in proteins [14], lipids [15], membrane [16], and DNA [17] of microorganisms. In addition, the mechanisms of disinfection are also highly dependent on the type of microorganism, cell growth stage, and disinfectant [18].

Other studies have considered the impact of disinfectants on *A. hydrophila* adhered to the fragments of polythene immersed in water. It appears that NaOCl is more effective on *A. hydrophila* adhered to polythene than H_2_O_2_. In addition, *A. hydrophila* adhered to polythene under dynamic condition is more sensitive to each of the two disinfectants than that adhered under static condition [18]. However, little data on the combined effect of these disinfectants are available. This study aims to evaluate in microcosm the synergistic effect of NaOCl and H_2_O_2_ on *A. hydrophila* cells from different cell growth phases and adhered to fragments of polythene immersed in water.

## 2. Materials and Methods

### 2.1. Collection and Identification of *A. hydrophila*

The bacterium *A. hydrophila* was isolated from well water in Yaoundé (Cameroon) using membrane filtration technique, on ampicillin-dextrin agar medium [19, 20]. Cell subculture was performed on standard agar medium (Bio-Rad Laboratories, France). The cells were then identified using standard biochemical methods [21]. These cells are facultative anaerobic, nonsporulated, Gram-negative bacilli, and ferment mannitol, produce indole, and are mobile. They do not possess urease, lysine decarboxylase (LDC), ornithine decarboxylase (ODC), and arginine dihydrolase (ADH). For the preparation of stocks of bacteria, colonies are inoculated into 100 mL of nutrient broth (Oxford) for 24 hours at 37°C. Afterwards, cells were harvested by centrifugation at 8000 rpm for 10 min at 10°C and washed twice with NaCl (8.5 g/L) solution. The pellet was resuspended in NaCl (8.5 g/L) solution and then transferred to 300 *μ*L tubes. The stocks were then frozen stored.

### 2.2. Assessment of Cell Growth Phase

On the basis of previous studies regarding the different growth phases and biofilm formation, the cell growth phases were assessed at 37°C. The growth of *A. hydrophila* in nonrenewed peptone liquid medium gives 4 growth phases: a lag growth phase from 0 to 2 hours, an exponential growth phase from 2 to 13 hours, a stationary growth phase from 13 to 22 hours, and a decline growth phase which begins as from the 22th hour [18].

### 2.3. Disinfectants and Adsorbent Substrates Used

The mixture of two disinfectants was used: NaOCl, which belongs to the group of halogen derivatives, and H_2_O_2_ which belongs to the group of oxidants. NaOCl and H_2_O_2_ used are, respectively, Colgate-Palmolive (USA) and Gilbert (France) brand. The ease use of these two disinfectants in drinking water treatment justified their choice for this study. The combination concentrations of each disinfectant used ranged from 0.1‰ to 0.3‰ and from 0.5‰ to 1.5‰, for NaOCl and H_2_O_2_, respectively. These concentrations were evaluated by simple method of dilution of crude solution obtained directly from the supplier. The choice of these combination concentrations is justified by their synergistic action. To count the surviving bacteria after disinfection treatment, sterile NaCl solution (8.5 g/L) was used as a diluent.

The substrate used is high dense polythene. It differs from radical low dense polythene and linear low dense polythene by the molecular structure of its sparsely branched chains and its relatively high resistance to shocks, high temperatures, and ultraviolet rays [22, 23]. It is a plastic piping material obtained directly from the supplier and used in drinking water distribution.

The high dense polythene is obtained by polymerization of the macromolecules of polyolefin family. This polymerization is obtained from gaseous ethylene according to the following equation [24, 25]:

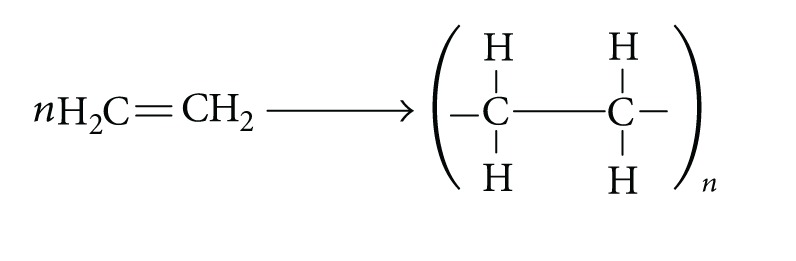
(9)


The polythene used in this study is commercialized by Goodfellow SARL (France).

### 2.4. Determination of Activity of Disinfectants Alone or in Combinations

The protocol described by Maris [26] with some modifications was applied. The principle of this protocol consists in preparation of the mixtures of NaOCl (A (B assoc)) and H_2_O_2_ (B (A assoc)). For it, nine couples of disinfectant concentrations (A (B assoc), B (A assoc)) were studied simultaneously for the preparation of mixtures of disinfectants. The disinfectant concentrations used alone ranged from 0.5‰ to 1.5‰ and from 5‰ to 15‰ for NaOCl (A alone) and H_2_O_2_ (B alone), respectively. The contaminated substrates are getting in contact with these disinfectant concentrations for 25 to 30 min. The disinfecting effect was stopped by introducing substrates in 10 mL of sterile saline. Antimicrobial activity was assessed after culture of surviving germs and appreciation of the reduction of the bacterial load.

The effect of the association was estimated by calculating the fractional bactericidal concentration (FBC) according to Maris [26]:

(10) $$\begin{array}{c} \text{FBC}=\frac{\text{A}\left(\text{B}\;\text{assoc}\right)}{\text{A}\left(\text{alone}\right)}+\frac{\text{B}\left(\text{A}\;\text{assoc}\right)}{\text{B}\left(\text{alone}\right)}, \end{array}$$

wherein A (B assoc) and B (A assoc) are the respective concentrations of NaOCl and H_2_O_2_ studied in the mixture. A (alone) and B (alone) are the respective concentrations of the two disinfectants studied alone.

The synergy was then declared for a value of FBC less than or equal to 0.50. The study of this synergy was achieved at each stage of cell growth phase in stationary and dynamic regimes.

### 2.5. Adhesion Protocol of Cells to Polythene

On the basis of previous studies, parallelepiped shaped fragments of polythene with 13.28 cm² of total surface area suspended with wire of 0.1 mm diameter were immersed in triplicate in the two sets A and B each in four flasks 250 mL Duran A1, A1′, and A1′′ and B1, B1′, and B1′′, A2, A2′, and A2′′ and B2, B2′, and B2′′, A3, A3′, and A3′′ and B3, B3′, and B3′′, and A4, A4′, and A4′′ and B4, B4′, and B4′′ each containing 99 mL of NaCl solution (8.5 g/L). Meanwhile, the controls were made and coded A_0_1, A_0_2, A_0_3, and A_0_4 and B_0_1, B_0_2, B_0_3, and B_0_4 [27]. The whole was then autoclaved.

Prior to the experiments, stocks frozen vial containing *A. hydrophila* cells were thawed at room temperature. Then 100 *μ*L of the culture was transferred into test tubes containing 10 mL of nutrient broth (Oxford) and incubated at 37°C for 24 hours. Cells from a specific cell growth phase were then harvested by centrifugation at 8000 rpm for 10 min at 10°C and washed twice with sterile NaCl solution (8.5 g/L). The pellets were then resuspended in 50 mL of sterilized NaCl solution (8.5 g/L). After serial dilutions, the initial concentration of bacteria (data at *t* = 0) in each solution was adjusted to 6 × 10^8^ CFU/mL by reading the optical density at 600 nm using a spectrophotometer (DR 2800) followed by culture on agar [27].

1 mL of the suspension was added to 99 mL of sterilized NaCl solution (8.5 g/L) contained in an Erlenmeyer flask. Triplicate flasks were incubated under dynamic condition for 180, 360, 540, and 720 min at a stirring speed of 60 rev/min, using a stirrer (Rotatest brand). In the same way another triplicate flasks were incubated under static condition for 180, 360, 540, and 720 min. All these incubations were done at laboratory temperature (25 ± 1°C).

### 2.6. Disinfection Experiments

After each incubation duration, fragments of polythene were drained for 10 seconds in a sterile environment created by the Bunsen burner flame and then introduced into test tubes containing 10 mL of diluted mixture of disinfectant of various concentrations. Fragments removed from flasks A1, A2, A3, A4, B1, B2, B3, and B4 were introduced in mixture disinfectant solutions of 0.1‰ NaOCl and 0.5‰ H_2_O_2_. Fragments removed from flasks A1′, A2′, A3′, A4′, B1′, B2′, B3′, and B4′ were introduced into mixture disinfectant solutions of 0.2‰ NaOCl and 1‰ H_2_O_2_. Similarly, those removed from flasks A1′′, A2′′, A3′′, A4′′, B1′′, B2′′, B3′′, and B4′′ were introduced into mixture solutions of 0.3‰ NaOCl and 1.5‰ H_2_O_2_. Fragments of polythene flasks from A_0_1, A_0_2, A_0_3, and A_0_4 and B_0_1, B_0_2, B_0_3, and B_0_4 were introduced into 10 mL of sterile NaCl solution (8.5 g/L). The concentration of the disinfectant has not been evaluated after incubation.

After 30 min of incubation at room temperature and under static condition, each fragment was then drained out under sterile condition. Each fragment was then introduced into 10 mL of sterilized NaCl solution (8.5 g/L). The unhooking of adherent cells was performed by vortex agitation at increasing speeds for 30 seconds in three consecutive series of 10 mL sterilized NaCl solution (8.5 g/L). This technique allows for the unhooking of maximum adhered cells [28, 29]. The total volume of the suspension containing the unhooked bacterial cells was 30 mL. The isolation and enumeration of unhooked cells were made by culture on ampicillin dextrin agar, by using spread plat method, followed by incubation on Petri dishes at 37°C for 24 hours.

### 2.7. Data Analysis

The variation of the abundance of adhered *A. hydrophila* in each experimental condition was illustrated by semilogarithmic diagrams. Standard deviations were not fitted because the curves were too close. Spearman “*r*” correlation Test was used to assess the degree of correlation between the abundance of adhered cells and other parameters considered. Kruskal-Wallis and Mann-Whitney tests were used to compare the mean abundance of cells adhered from one experimental condition to another.

The data from absorption experiments were analyzed using the Freundlich isotherm model. This isotherm was chosen because of the number and the relevance of the information it provides on the real adsorption mechanisms on one hand and its remarkable ability to match doses of adsorption on the other hand. The Freundlich isotherm is described by the following equation [30, 31]:

(11) $$\begin{array}{c} C_{s}=K_{f}\cdot C^{l/n}, \end{array}$$

where *C*_*s*_ is the quantity of cells adsorbed in the presence of the mixture of disinfectant solutions, *C* is the concentration of cells adsorbed in the absence of mixture of disinfectant solutions, *K*_*f*_ is the Freundlich coefficient adsorption which is connected to the adsorption capacity, *l*/*n* is coefficient linearity, and *n* is the intensity of adsorption. Here, *Cs* is expressed as the number of adherent cells/mixture of disinfectant concentration and *C* is the number of adherent cells/cm² of polythene. Constructing linear regression log *Cs* versus log *C* results in a line of slope *l*/*n* which intercepts the *y*-axis log *K*_*f*_.

## 3. Results

### 3.1. Fractional Bactericidal Concentration (FBC)

The FBC values were calculated using the formula indicated above. The different FBC obtained is given in Table 1. To ensure the synergistic action of the two disinfectants, only disinfectant concentrations giving FBC equal to 0.3 were used for the preparation of mixture of disinfectants.

### 3.2. Abundance of Cells Adhered to Polythene after Action of the Association of Disinfectants in Stationary Regime

**Figure 1:**
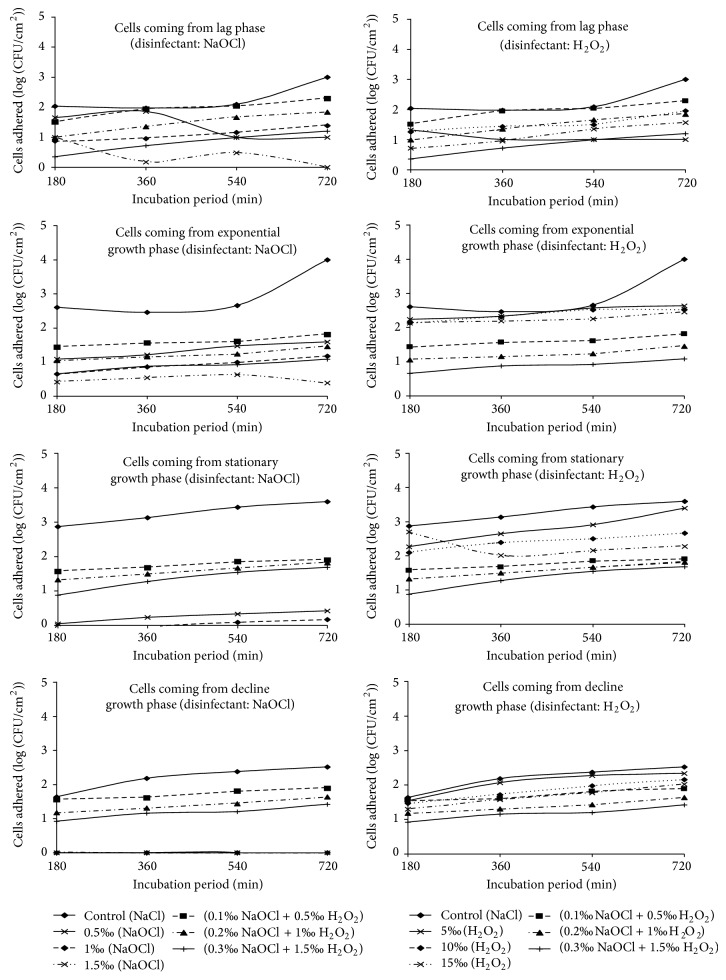
Temporal evolution of cells adhered under static condition after the action of NaOCl and H_2_O_2_ alone and in the mixture of the two disinfectants at different concentrations.

The densities of cells adhered ranged from 0.30 to 2.29 units (log (CFU/cm²)) after the action of the mixture of NaOCl and H_2_O_2_ under static condition. The maximum abundance of cells adhered was recorded in the presence of the mixture of 0.1‰ NaOCl and 0.5‰ H_2_O_2_ and this is after 720 minutes with cells harvested from the lag growth phase. Adhered cells were always partially decimated by the mixture of NaOCl and H_2_O_2_ (Figure 1).

With cells coming from the lag phase, the abundance of cells adhered under static condition to the control substrate varied throughout from 2.02 to 3.19 units (log (CFU/cm²)) and was always superior to those of fragments tested for disinfection. In addition, they increase with the incubation duration. Maximum cell density was recorded after an adhesion test of 720 minutes. After the action of the mixture of NaOCl and H_2_O_2_, the densities of cells adhered ranged from 0.30 to 2.29 units (log (CFU/cm²)). The effectiveness of the mixture of NaOCl and H_2_O_2_ decreased with the length of the adhesion duration test. The maximum cell abundance was recorded in the presence of the mixture of 0.1‰ NaOCl and 0.5‰ H_2_O_2_ after an adhesion test of 720 minutes. The lowest density of adhered cells was observed in the presence of the mixture of 0.3‰ NaOCl and 1.5‰ H_2_O_2_ with cells coming from the adhesion tests of 180 minutes (Figure 1).

The abundance of cells under static condition adhered to the control substrate during the exponential growth phase was lower than that tested for disinfection in the lag growth phase under the same condition. They generally fluctuated between 2.30 and 2.91 units (log (CFU/cm²)). After disinfection test, it was noted that the effectiveness of the mixture of NaOCl and H_2_O_2_ decreased when the duration of adhesion test increased. Abundance of cells adhered ranged between 0.70 to 1.81 units (log (CFU/cm²)) (Figure 1). The highest cell abundance was recorded in presence of the mixture of 0.1‰ NaOCl and 0.5‰ H_2_O_2_ after an adhesion test of 720 minutes. The lowest density of adhered cells was observed in the presence of the mixture of 0.3‰ NaOCl and 1.5‰ H_2_O_2_ with cells coming from the adhesion tests of 180 minutes (Figure 1).

The stationary growth phase shows the abundance of cells in static regime adhered to the control substrate which varies from 1.92 to 2.49 units (log (CFU/cm²)). They remained higher than those of the fragments tested for disinfection. After disinfection test, abundance of cells adhered ranged between 0.90 and 1.89 units (log (CFU/cm²)). As the duration of adhesion test increased, it was noted that the effectiveness of the mixture of NaOCl and H_2_O_2_ decreased. The highest density of cells adhered to the polythene was recorded in the presence of the mixture of 0.3‰ NaOCl and 1.5‰ H_2_O_2_ after 720 minutes incubation duration. The lowest density of adhered cells was observed in the presence of mixture of 0.3‰ NaOCl and 1.5‰ H_2_O_2_ after 180 minutes incubation duration (Figure 1).

The abundance of cells adhered in static regime to the control substrate during the decline growth phase varied from 1.95 to 2.48 units (log (CFU/cm²)). Adhered cells after the action of NaOCl relatively increased (Figure 1). The maximum density of cells adhered to the polythene was recorded in the presence of the mixture of 0.3‰ NaOCl and 1.5‰ H_2_O_2_ after 720 minutes incubation duration. The minimum density of adhered cells was observed in the presence of mixture of 0.3‰ NaOCl and 1.5‰ H_2_O_2_ after 180 minutes incubation (Figure 1).

### 3.3. Abundance of Cells Adhered to Polythene after Action of Association of Disinfectants in Dynamic Regime

The abundances of cells adhered ranged from 0.85 to 2.27 units (log (CFU/cm²)) after the action of the mixture of NaOCl and H_2_O_2_ under dynamic condition. The maximum abundance of cells adhered was recorded in the presence of mixture of 0.1‰ NaOCl and 0.5‰ H_2_O_2_ and this is after 720 minutes with cells harvested from the lag growth phase.

**Figure 2:**
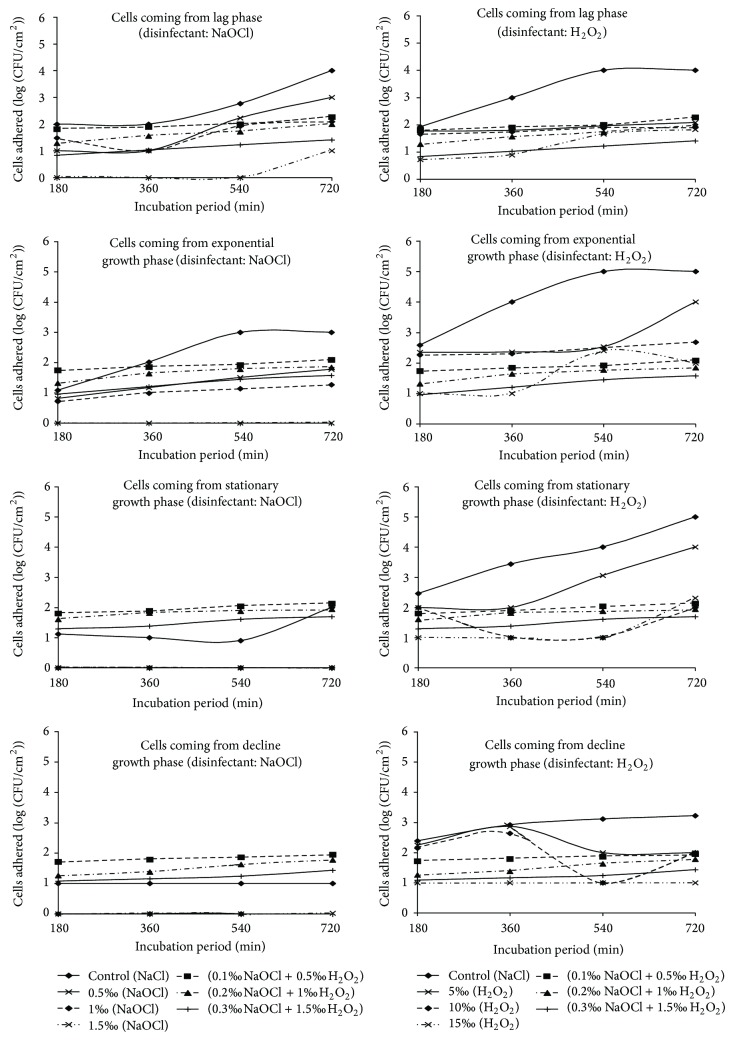
Temporal evolution of cells adhered under dynamic condition after the action of NaOCl and H_2_O_2_ alone and in the mixture of the two disinfectants at different concentrations.

The density of cells adhered under dynamic condition to the control substrate varied throughout from 2.35 to 3.25 units (log (CFU/cm²)) from the lag phase and was always superior to those fragments tested for disinfection. In addition, they increase with the incubation duration. The maximum cell abundance was recorded in the presence of the mixture of 0.1‰ NaOCl and 0.5‰ H_2_O_2_ after an adhesion test of 720 minutes. The lowest density of adhered cells was observed in the presence of the mixture of 0.3‰ NaOCl and 1.5‰ H_2_O_2_ with cells coming from the adhesion tests of 180 minutes (Figure 2). After action of the mixture of NaOCl and H_2_O_2_, the densities of cells adhered ranged from 0.85 to 2.27 units (log (CFU/cm²)). The effectiveness of the mixture of NaOCl and H_2_O_2_ decreased with the length of the adhesion test duration.

Abundance of cells adhered under dynamic condition to control substrate during the exponential growth phase was lower than that tested for disinfection in the lag growth phase under the same condition. They generally fluctuated between 2.47 and 3.19 units (log (CFU/cm²)). After disinfection test, it was noted that the effectiveness of the mixture of NaOCl and H_2_O_2_ decreased when the duration of adhesion test increased. Abundance of cells adhered ranged between 0.95 and 2.09 units (log (CFU/cm²)) (Figure 2). The maximum cell abundance was recorded in presence of mixture of 0.1‰ NaOCl and 0.5‰ H_2_O_2_ after an adhesion test of 720 minutes. The minimum density of adhered cells was observed in the presence of mixture of 0.3‰ NaOCl and 1.5‰ H_2_O_2_ with cells coming from the adhesion tests of 180 minutes (Figure 2).

The abundance of cells adhered in dynamic regime to the control substrate varied from 2.35 to 2.74 units (log (CFU/cm²)) during the stationary growth phase. It remained higher than those of fragments tested for disinfection. After disinfection test, abundance of cells adhered ranged between 1.30 and 2.13 units (log (CFU/cm²)). As the duration of adhesion test increased, it was noted that the effectiveness of the mixture of NaOCl and H_2_O_2_ decreased. The maximum density of cells adhered to the polythene was recorded in the presence of the mixture of 0.3‰ NaOCl and 1.5‰ H_2_O_2_ after 720 minutes incubation duration, whereas the minimum density was observed in the presence of the mixture of 0.3‰ NaOCl and 1.5‰ H_2_O_2_ after 180 minutes incubation duration (Figure 2).

Density of cells adhered in dynamic condition to the control substrate during the decline growth phase varied from 2.10 to 2.71 units (log (CFU/cm²)). Cells adhered after the action of NaOCl were relatively high (Figure 2). The maximum density of cells adhered to the polythene was recorded in the presence of the mixture of 0.3‰ NaOCl and 1.5‰ H_2_O_2_ after 720 minutes incubation duration and the minimum in the presence of the mixture of 0.3‰ NaOCl and 1.5‰ H_2_O_2_ after 180 minutes incubation (Figure 2).

### 3.4. Freundlich Isotherms of Cells Adsorption

**Figure 3:**
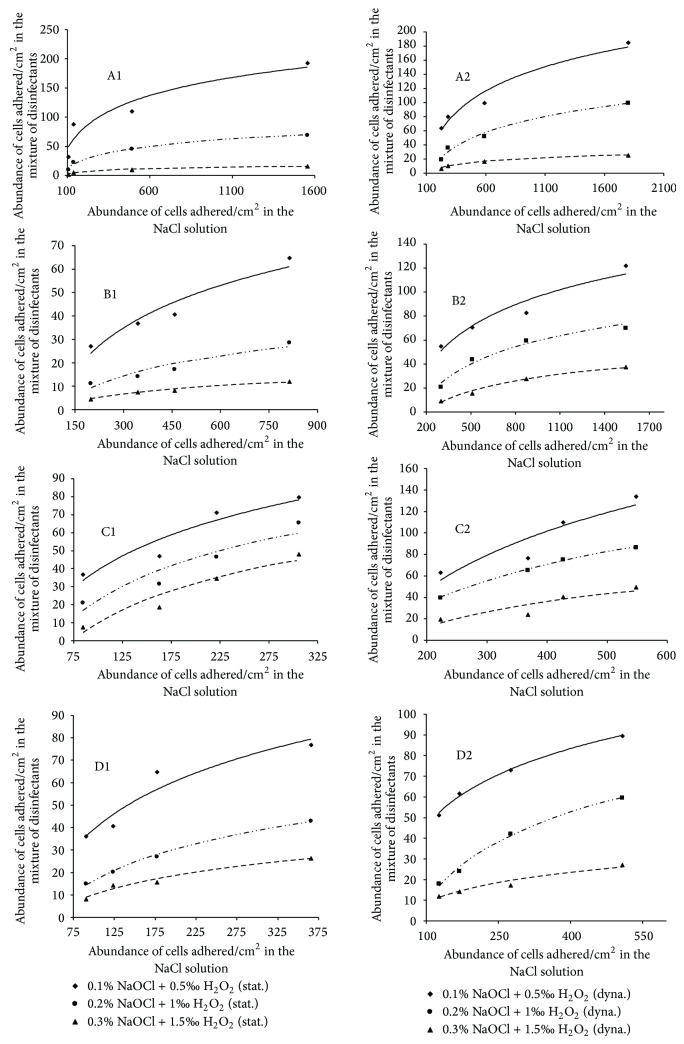
Freundlich isotherms for cells absorption under static (A1, B1, C1, and D1) and dynamic (A2, B2, C2, and D2) conditions in the presence of the mixture of NaOCl and H_2_O_2_ (lag growth phase (A1, A2), exponential growth phase (B1, B2), stationary growth phase (C1, C2), and decline growth phase (D1, D2)).

Freundlich isotherms were constructed by considering only the combination concentrations, the number of cells adhered to the substrate, subjected to the test of disinfection, and obtained without exposure to the mixture of disinfectants for each stage of cell growth and each experimental condition. The Freundlich isotherms are shown in Figure 3. It can be noted that, no matter which growth stage cells are, the appearance of the isotherms differs from one incubation condition to another. The linearity coefficient *l*/*n* which is related to the adsorption intensity ranged from 0.01 to 0.21 and from 0.02 to 0.15, respectively, under static and dynamic incubation conditions. The adsorption coefficient *K*_*f*_ which is related to the adsorption capacity ranged between 2 and 53 and between 2 and 54 cells adhered, respectively, under static and dynamic incubation conditions. The adsorption coefficient for the lag growth phase ranged between 4 and 53 and between 2 and 54 cells adhered, respectively, under static and dynamic conditions (Table 2). The lowest adsorption coefficient after the mixture of disinfectant treatment was obtained with cell harvested from the lag growth phase (Table 2).

When considering each experimental condition, the adsorption coefficient of cells harvested from the lag phase was relatively higher after the mixture of disinfectant treatment than that of cell harvested from the other cells growth phases (Table 2). It was also noted that for the whole cell growth phases and the whole incubation conditions, the adsorption coefficient values were relatively higher with the mixture of 0.1‰ NaOCl and 0.5‰ H_2_O_2_ concentration than those of the two other mixture of disinfectant concentrations (Table 2).

### 3.5. Correlation Coefficients between the Abundance of Cells Adhered and Incubation Durations and Concentrations of Disinfectants

Spearman “*r*” correlation coefficients between the abundances of cells adhered and incubation durations for each concentration of mixture of disinfectant and each experimental condition were assessed and are presented in Table 3. It is noted that the increase in the incubation durations caused a significant decrease in the efficiency of 0.3‰ NaOCl and 0.3‰ H_2_O_2_ mixture of disinfectant concentration (*P* < 0.01). This could result in higher abundance of cells adhered as the duration of the cell adhesion process increased.

Spearman “*r*” correlation coefficients between abundance of cells adhered and concentrations of the mixture disinfectants for each incubation duration and under each experimental condition were also assessed (Table 4). Under static as well as dynamic condition, it was noted that the effectiveness of the mixture of disinfectant concentrations on cells adhered to polythene increased leading to a significant decrease (*P* < 0.01) in the abundance of bacteria adhered after disinfection treatment.

The degrees of relationship between the mixture of disinfectant concentrations and abundance of cells adhered harvested from each growth stage were also assessed (Table 5). It resulted that an increase in the mixture of disinfectant concentration significantly increased (*P* < 0.01) the abundance of cells adhered to the substrate, with cell harvested from each cell growth phase.

### 3.6. Comparison of the Mean Abundance of Cells Adhered amongst the Different Stages of Cell Growth

The *H* test of Kruskal-Wallis was performed in order to compare the mean abundance of cells adhered harvested from different cell growth stages and considering each mixture of disinfectants concentrations. It showed that there is an overall significant difference (*P* < 0.05) between the mean abundance of cells adhered to polythene for each mixture of disinfectant concentration at different cell growth stages. The pair two-by-two comparisons of the mean abundances were then performed using the *U* test of Mann-Whitney. It was noted that, at each cell growth stage, there was a significant difference (*P* < 0.05) amongst the mean abundance of cells adhered after the action of various mixture of disinfectant concentrations with cells coming from each cell growth phase. With the mixture of 0.1‰ NaOCl and 0.5‰ H_2_O_2_ and that of 0.3‰ NaOCl and 1.5‰ H_2_O_2_, a nonsignificant difference was observed only with cells harvested from the stationary cell growth phase (*P* ≥ 0.05) (Table 6).

## 4. Discussion

The aim of this study was to determine the synergistic effect of NaOCl and H_2_O_2_ on *A. hydrophila* adhered to polythene immersed in water under static and dynamic conditions. By contrast, most previous studies have indicated only the effect of NaOCl on one hand and that of H_2_O_2_ on the other hand on the adhesion of *A. hydrophila* to polythene [18, 32, 33]. From the 9 pairs of concentration of disinfectants used for the preparation of mixture of disinfectants, three couples (0.1‰ NaOCl + 0.5‰ H_2_O_2_; 0.2‰ NaOCl + 1‰ H_2_O_2_; and 0.3‰ NaOCl + 1.5‰ H_2_O_2_) were used to evaluate the synergy as they presented an FBC equal to 0.3. A synergy is declared when a value of FBC is less than or equal to 0.50 [26].

The present study showed that the overall abundance of cells adhered to polythene after the action of the mixture of two disinfectants was lower than that obtained after the action of H_2_O_2_ alone. Abundance of cells adhered to polythene ranged from 0.30 to 2.29 and 0.85 to 2.27 units (log (CFU/cm²)) after the action of the mixture of NaOCl and H_2_O_2_ under static and dynamic conditions, respectively. Previous studies showed that they sometimes reached 2.41 and 3.39 units (log (CFU/cm²)) after the action of NaOCl and H_2_O_2_, respectively [18]. These results suggest that the combination of NaOCl and H_2_O_2_ leads to a significant synergy in eliminating cells adhered to polythene. This has been also suggested in previous studies [34].

Abundance of cells adhered to polythene after the action of the mixture of NaOCl and H_2_O_2_ was relatively higher than those obtained after the action of NaOCl alone. The maximum abundance of cells adhered to polythene was recorded under static condition in the presence of the mixture of 0.1‰ NaOCl and 0.5‰ H_2_O_2_ and this is after 720 minutes with cells obtained in the lag growth phase (Figures 1 and 2). That obtained after the action of NaOCl was recorded during the lag phase under dynamic condition in the presence of 0.5‰ concentrations of NaOCl and this is after an adhesion test of 720 minutes. By cons, the abundance of cells adhered to polythene after the action of the mixture of NaOCl and H_2_O_2_ was considerably lower than those obtained after the action of H_2_O_2_.

The maximum abundance of cells adhered after the action of H_2_O_2_ was recorded during the stationary growth phase under static condition in the presence of 5‰ H_2_O_2_ concentration after the same period of adhesion test. Due to its highly oxidizing capacity-based production of free radicals that affect the biofilms matrix H_2_O_2_ was chosen to fight effectively against biofilms formation [35, 36]. In addition, H_2_O_2_ was chosen as it is highly effective disinfectant in inhibiting biofilms formation at a concentration of 0.05%. It can also destroy mature biofilms at concentrations between 0.08% and 0.2% [37]. The reaction between NaOCl and H_2_O_2_ produces singlet oxygen (^1^O_2_), which is a powerful oxidant that rapidly kills bacterial cells. In addition, oxygen singlet short lifespan (100 nanoseconds in lipid media and 50 nanoseconds in the cytoplasm) can diffuse a short distance and react with certain amino acids leading to structural and functional alteration of the membrane causing lipoperoxidation [13]. NaOCl and H_2_O_2_ inhibit the Brownian motion and control the growth of the microbial population [34].

The adhesion of microorganisms to surfaces is the first step in biofilms formation, which is a form of microbial life in aquatic environments [38]. The latter is the source of problems bioburden in various fields such as health, environment, food industry, and water purification [31, 39, 40]. Adhesion is governed by physicochemical interactions of the Van Der Waals and Lewis acid-base types. Fluctuating velocities of adhesion of cells observed during different stages of growth in stationary and dynamic regimes could be explained by changes in the physiology of bacterium at each stage of growth [41, 42]. There are three strategies against biofilms formation: (i) the disinfection time before the biofilms develop, (ii) the disinfection of biofilms using aggressive disinfectants, and (iii) inhibition fixing microbes choosing surface materials that do not promote adherence [43].

By considering separately each condition, it was noted that the increase in incubation durations resulted in a significant decrease (*P* < 0.01) in the effectiveness of the mixture of 0.3‰ NaOCl and 1.5‰ H_2_O_2_ (Table 3). This resulted in higher abundance of cells. Indeed, a biofilm can be developed within in a few hours, allowing bacteria therein to become resistant to external agents causing any contamination [44, 45]. In static as well as dynamic condition, increasing the effectiveness of the mixture concentration of NaOCl and H_2_O_2_ on cells adhered to polythene resulted in a significant decrease in abundance of cells adhered after disinfection test (*P* < 0.01) (Figures 1 and 2). The treatment of biofilms by combining antimicrobial agents has a synergistic effect on the removal of adherent bacterial cells [34]. Furthermore, this variation of the reaction of cells against the combination of disinfectants may be related to changes in the surface due to a change in their growth phase [46].

It was also noted that for each incubation period and each cell growth phase, a rise in the concentration of disinfectant mixture increases significantly (*P* < 0.01) the abundance of cells adhered to the substrate (Table 4). Face with antimicrobial agent bacteria develops biofilm formation as a coping strategy [47, 48]. For each cell growth phase, a significant difference was observed between the mean densities of cells adhered after the action of the different concentrations of the mixture of disinfectants (*P* < 0.05). The effectiveness of any method of disinfection depends on biotic factors such as the physiological state and the intrinsic microbial resistance to lethal agents [49]. The age of the culture also plays an important role since the adhesion of the bacterium is better during exponential growth phase than stationary growth phase [50].

It is important to remember that bacteria in a biofilm have very different characteristics from their planktonic counterparts including the production of exopolymers [51], a significant increase in antimicrobial resistance and environmental stress [52, 53]. The matrix of exopolymers which presents itself as a mechanical barrier, reducing the penetration of environmental compounds through the biofilms, thus protects bacterial cells embedded in biofilm. This explains the fact that the increase in the concentration of the mixture of disinfectants for each stage of growth leads to a significant increase (*P* < 0.01) in abundance of cells adhered to the substrates. The adsorption coefficient (*K*_*f*_) was relatively higher in the static than in the dynamic regime no matter the cell growth phase or presence of a well-defined concentration of the mixture of disinfectant. Cells adhered to polythene under dynamic condition were more sensitive than that obtained with the two combined disinfectants under static condition. This could be explained by the structure of adhered bacteria which depends on the hydrodynamic regime [54]. Enzymes produced by *A. hydrophila* are essentially proteases, esterases, and lyases. Although these enzymes often remain qualitatively unchanged with bacterial growth phase [55], they would quantitatively be modified from one cell growth stage to another.

## 5. Conclusion

This study showed that the combination of NaOCl and H_2_O_2_ has a synergistic effect on cells adhered to polythene. Abundance of cells adhered to polythene after the action of the mixture of NaOCl and H_2_O_2_ is relatively higher than that obtained after the action of NaOCl alone. By cons, it is significantly lower than that obtained after the action of H_2_O_2_ alone. Under static as well as dynamic condition, an increase in the effectiveness of the concentrations of the mixture of NaOCl and H_2_O_2_ on cells adhered is noted. For each cell growth phase, the densities of cells adhered differed from a given concentration of a mixture of disinfectants to another. Although the adsorption coefficient (*K*_*f*_) obtained from the Freundlich isotherm is relatively higher in static state than in dynamic regime, cells adhered to polythene in the presence of the mixture of the two disinfectants under dynamic condition seem more sensitive than under static condition.

## Figures and Tables

**Table 1:** Value of fractional bactericidal concentration (FBC) obtained for each couple of disinfectants concentrations.

Concentrations of disinfectant used				FBC
Disinfectants in mixture		Disinfectants alone	
NaOCl (‰)	H_2_O_2_ (‰)	NaOCl (‰)	H_2_O_2_ (‰)
0.1	0.5	0.5	5	0.3
0.2	1	1	10	0.3
0.3	1.5	1.5	15	0.3
0.1	2	0.5	5	0.6
0.2	3	1	10	0.5
0.3	4	1.5	15	0.46
0.25	5	0.5	5	1.5
0.5	6	1	10	1.1
0.75	8	1.5	15	1.03

**Table 2:** Values of adsorption coefficient (*K*_*f*_) (adhered *A. hydrophila*/mL of mixture of disinfectant) and linearity coefficient (*l*/*n*) of isotherms under static and dynamic conditions, when using different disinfectants concentrations.

Disinfectant concentrations and static or dynamic condition		Freundlich isotherm coefficients according to the cell growth phase							
		Adsorption coefficient (cells adhered/cm²)				Linearity coefficient			
Disinfectant concentrations	Condition	Lag	Expo.	Stat.	Decl.	Lag	Expo.	Stat.	Decl.
0.1‰ NaOCl + 0.5‰ H_2_O_2_	Static	53	15	18	27	0.09	0.06	0.21	0.14
	Dynamic	54	41	7	44	0.07	0.05	0.02	0.09

0.2‰ NaOCl + 1‰ H_2_O_2_	Static	16	5	2	8	0.04	0.03	0.20	0.10
	Dynamic	3	20	10	7	0.05	0.04	0.15	0.11

0.3‰ NaOCl + 1.5‰ H_2_O_2_	Static	4	3	9	5	0.01	0.01	0.19	0.06
	Dynamic	2	4	5	7	0.05	0.02	0.10	0.04

**Table 3:** Spearman “*r*” correlation coefficients between the abundances of adhered* A. hydrophila* and incubation durations for each concentration of mixture of disinfectant and each experimental condition.

Experimental condition	Mixtures of disinfectant concentrations		
	0.1‰ NaOCl + 0.5‰ H_2_O_2_	0.2‰ NaOCl + 1‰ H_2_O_2_	0.3‰ NaOCl + 0.3‰ H_2_O_2_
Static	0.800	−0.200	−0.400^**^
Dynamic	0.400	0.632	−0.949^**^

^**^ *P* < 0.01; ddl = 15.

**Table 4:** Spearman “*r*” correlation coefficients between the abundance of adhered* A. hydrophila* and concentration of mixture of disinfectant for each incubation duration and under each experimental condition.

Experimental condition	Incubation durations			
	180 min	360 min	540 min	720 min
Static	1.000∗∗	1.000∗∗	1.000∗∗	1.000∗∗
Dynamic	1.000∗∗	1.000∗∗	1.000∗∗	1.000∗∗

^**^ *P* < 0.01; ddl = 15.

**Table 5:** Spearman “*r*” correlation coefficients between the abundance of adhered* A. hydrophila* and incubation durations for each concentration of the mixture of disinfectant and each cell growth phase.

Cell growth phase	Mixtures of disinfectant concentrations		
	0.1‰ NaOCl + 0.5‰ H_2_O_2_	0.2‰ NaOCl + 1‰ H_2_O_2_	0.3‰ NaOCl + 0.3‰ H_2_O_2_
Lag	0.947∗∗	0.950∗∗	0.981∗∗
Exponential	0.970∗∗	0.964∗∗	0.905∗∗
Stationary	0.955∗	0.920∗∗	0.694∗∗
Decline	0.980∗∗	0.930∗∗	0.945∗∗

^**^ *P* < 0.01; ^*^ *P* < 0.05; ddl = 31.

**Table 6:** Comparison amongst abundance of *A. hydrophila* harvested from different cell growth stages in the presence of each mixture of disinfectant concentrations.

Cell growth phase	Mixtures of disinfectant concentrations		
	0.1‰ NaOCl + 0.5‰ H_2_O_2_	0.2‰ NaOCl + 1‰ H_2_O_2_	0.3‰ NaOCl + 1.5‰ H_2_O_2_
Lag	*P* = 0.015^*^	*P* = 0.000^*^	*P* = 0.005^*^
Exponential	*P* = 0.050^*^	*P* = 0.001^*^	*P* = 0.038^*^
Stationary	*P* = 0.161	*P* = 0.003^*^	*P* = 0.065
Decline	*P* = 0.007^*^	*P* = 0.000^*^	*P* = 0.021^*^

^*^ *P* < 0.05; ddl = 92.
